# Dicer Neosynthesis Regulates Platelet Reactivity: A Mechanism Altered in Type 2 Diabetes

**DOI:** 10.1161/CIRCRESAHA.124.325357

**Published:** 2025-08-05

**Authors:** Loredana Bury, Elisa Piselli, Giorgia Manni, Stefania Momi, Giulia Ciarrocca Taranta, Giuseppe Guglielmini, Carmine Fanelli, Michelantonio De Fano, Jesse W. Rowley, Andrew S. Weyrich, Paolo Gresele

**Affiliations:** Division of Internal and Cardiovascular Medicine (L.B., E.P., G.M., S.M., G.C.T., G.G., P.G.), University of Perugia, Italy.; Division of Endocrino-Metabolic Sciences, Department of Medicine and Surgery (C.F., M.D.F.), University of Perugia, Italy.; Department of Internal Medicine, University of Utah School of Medicine, Salt Lake City (J.W.R., A.S.W.).

**Keywords:** gene expression, megakaryocytes, microRNAs, platelet activation

## Abstract

**BACKGROUND:**

Despite being anucleate, platelets contain mRNAs and synthesize new proteins. Platelets also contain microRNAs and Dicer (ribonuclease III enzyme Dicer-1), an enzyme required for microRNA maturation. The expression of Dicer and some microRNAs is reduced in platelets from patients with type 2 diabetes (T2DM). However, the role of Dicer in the regulation of platelet function and in T2DM-associated platelet hyperreactivity is unclear. We aimed to assess whether Dicer levels are regulated in platelets upon activation, if they modulate mRNA translation by triggering pre-microRNA maturation, and whether these mechanisms are deranged in T2DM platelets.

**METHODS:**

Dicer expression in ultrapurified platelets was assessed by Western blotting, flow cytometry, and liquid chromatography-tandem mass spectrometry. P2Y_12_ (purinergic receptor P2Y_12_) expression was assessed by Western blotting and microRNA-223 and P2RY12 transcript by real-time polymerase chain reaction. In vivo experiments were performed in Dicer-deficient and wild-type mice with alloxane-induced diabetes.

**RESULTS:**

Thrombin-activated platelets from healthy individuals rapidly neosynthesize Dicer, leading to increased maturation of microRNA-223 and concomitant consumption of pre-microRNA-223. An increase in microRNA-223 was associated with a reduction of P2RY12 mRNA, one of its main targets, and platelet P2Y_12_ expression and function. All these mechanisms were significantly deranged in platelets from patients with T2DM. Similar alterations were also observed in platelets from Dicer-deficient and diabetic mice. ADP-induced platelet pulmonary thromboembolism was decreased in mice previously infused with low-dose thrombin.

**CONCLUSIONS:**

The Dicer-triggered decrease in P2Y_12_ after thrombin stimulation may represent a self-regulatory mechanism of platelet activation to prevent undesired thrombus formation. The derangement of this self-regulatory mechanism in T2DM may contribute to the platelet hyperreactivity and enhanced thrombotic complications of patients with T2DM. Our results show that the complex regulatory role of microRNA neoformation during platelet activation, when deranged, may contribute to cardiovascular disease.

**Novelty and Significance**

**What Is Known?**

- Platelets contain microRNAs and the enzyme Dicer (ribonuclease III enzyme Dicer-1), which transforms inactive precursor microRNAs into their active mature forms.
- In type 2 diabetes (T2DM), platelet microRNA-223 levels and Dicer protein levels are reduced.
- The expression of P2Y_12_ (purinergic receptor P2Y_12_), a platelet receptor mediating ADP-induced activation, is regulated by microRNA-223 and is hyperexpressed in T2DM.

**What New Information Does This Article Contribute?**

- Platelets rapidly synthesize new Dicer upon thrombin stimulation, triggering the maturation of microRNA-223 and downregulation of P2Y_12_.
- This mechanism acts as a self-regulatory pathway downregulating platelet activation and thrombus formation.
- In T2DM, this pathway is dysfunctional, contributing to platelet hyperreactivity and a prothrombotic state.

Although platelets lack a nucleus, they are able to synthesize certain proteins in response to stimuli. This study demonstrates for the first time that platelets rapidly produce the microRNA-processing enzyme Dicer upon thrombin stimulation. This neosynthesis leads to the generation of mature, active microRNA-223, which, in turn, downregulates its target, the mRNA for P2RY12 (purinergic receptor P2Y_12_). P2RY12 codes for the ADP receptor P2Y_12_, activation of which leads to increased thrombosis. Thus, downregulation of P2Y_12_ levels dampens platelet hyperactivity. This microRNA-223-P2RY12 self-regulatory mechanism is meant to prevent excessive platelet activation and thrombus formation in vivo. The microRNA-223-P2RY12 self-regulatory pathway is disrupted in platelets from patients with T2DM, leading to insufficient microRNA-223 maturation and impaired downregulation of P2Y_12_. These findings provide new insights into the molecular regulation of platelet activation and may help explain the enhanced thrombotic risk in T2DM. The discovery of Dicer neosynthesis as a key checkpoint in platelet reactivity expands our understanding of posttranscriptional gene regulation in anucleate platelets and highlights potential targets for therapeutic intervention in thromboinflammatory conditions such as diabetes.

**Meet the First Author, see p 810**

Platelets harbor an abundant and diverse array of microRNAs^1^ and short noncoding RNA species, which regulate posttranscriptional gene expression by degrading or inhibiting specific target mRNAs. In mammalian cells, microRNAs are initially transcribed as longer molecules (pri-microRNAs), which are then processed in the nucleus into hairpin precursor microRNAs (pre-microRNAs) by the protein Drosha. Pre-microRNAs are then transported to the cytoplasm where they are digested by a second, double-strand–specific ribonuclease called Dicer1 (ribonuclease III enzyme Dicer-1), a cytoplasmic RNase III enzyme. Mature microRNAs are then incorporated into a ribonucleoprotein complex (RISC [RNA-induced silencing complex]) that downregulates target gene activity, resulting in reduced levels of the corresponding transcripts.^2^ Platelets contain the cytoplasmic enzymes of the RNA interference machinery, including Dicer1, and can, thus, process megakaryocyte-derived pre-microRNAs into mature microRNAs.^1^

The role and importance of microRNAs in platelets have emerged over the past decade with evidence that they regulate protein translation and platelet reactivity.^3^ In particular, microRNA-223, the most abundant in platelets, has P2RY12 (purinergic receptor P2Y_12_) mRNA among its main targets^1^ with its translation product P2Y_12_ being one of the crucial receptors regulating platelet reactivity. microRNA profiling studies have revealed that type 2 diabetes (T2DM) is associated with decreased levels of several platelet microRNAs^4–6^ and, in particular, microRNA-223, with consequently increased expression of platelet P2RY12 mRNA and P2Y_12_ protein contributing to platelet hyperreactivity.^7,8^ Dicer1 is reduced in diabetic platelets due to its cleavage by calpain, which is hyperactivated in diabetes.^9^

The microRNA expression pattern is modified upon platelet activation in vitro^3,10^ and in vivo,^11,12^ and platelet miRNome changes are, in turn, associated with proteome modifications.^13^ All these data suggest that changes in the microRNA-processing enzymes may regulate platelet reactivity in health and disease; however, the mechanisms driving these changes have not been fully clarified.

Despite the lack of a nucleus, circulating platelets synthesize new proteins in response to activation in a rapid and sustained fashion, thanks to the translation of the mRNAs that they inherit from megakaryocytes.^14,15^ It is, thus, conceivable that activated platelets may neosynthesize Dicer1. Key regulators of mRNA translation and, thus, protein synthesis in platelets are mTOR (mammalian target of rapamycin)^16^ and Mnk1 (mitogen-activated kinase kinase-interacting kinase 1).^17^

Based on these premises, we aimed to assess whether neosynthesis of Dicer1 occurs in platelets upon activation, whether this is regulated by mTOR and Mnk1, and if this has an impact on platelet microRNA and mRNA levels, thus regulating the expression of specific target proteins, and to evaluate whether these mechanisms are deranged in platelets from patients with T2DM.

## Methods

### Data Availability

The data that support the findings of this study are available from the corresponding author upon request.

### Study Approval

All the studies were approved by the institutional review boards: the Comitato Etico Aziende Sanitarie of Umbria CEAS (approval number 2663/15) for studies involving humans and the Italian Ministry of Public Health (authorization number 561/2015-PR) for studies involving mice.

### Patients and Healthy Controls

Thirty-seven male and female patients with T2DM were recruited at the Diabetology Clinic of the University of Perugia, and blood samples were taken during visits scheduled for clinical follow-up. Thirty-seven age- and sex-matched healthy controls were recruited among laboratory staff and contacts. A detailed description of inclusion and exclusion criteria is reported in the Supplemental Material.

### Animals

Dicer1^*Pf4Δ/Pf4Δ*^ mice were generated by gene targeting, as reported. Briefly, mice harboring a floxed allele of Dicer1^18^ were crossed to animals expressing Cre-recombinase controlled by the Pf4 promoter^19^ to generate Dicer1^*Pf4Δ/Pf4Δ*^ mice lacking Dicer1 specifically in megakaryocytes and platelets. Dicer1^*fl/fl*^ (B6.Cg-Dicer1tm1Bdh/J) mice were used as controls.^18^ Wild-type C57BL/6 mice were purchased from Charles River (Calco, Italy) and studied at the age of 8 weeks.

C57BL6 mice were made diabetic through the intravenous injection of alloxan monohydrate (Sigma A7413; 60 mg/kg) dissolved in PBS. Mice were considered diabetic when glycemia rose to 20 mmol/L.^20^

Dicer1^*Pf4Δ/Pf4Δ*^ mice were housed at the University of Utah, the Walter and Eliza Hall Institute, Salt Lake City, UT; C57BL6 mice were housed at the University of Perugia, Center for Preclinical Research, Perugia, Italy. Eight-week-old male mice were randomly distributed in cages and treated sequentially. Blinding was performed at the time of allocation of animals to treatment groups. Moreover, the investigator who analyzed the data was not aware of which treatment each group received. In experiments in which mice were made diabetic, those with a glycemia <20 mM were excluded.

### Studies in Mice

#### In Vivo Stimulation With Thrombin

Human thrombin (Sigma, T6884) was injected in mice into the tail vein at a dose of 500 U/kg (≈10 U/mouse) in a fixed volume of 100 µL. This is a sublethal dose for mice when injected in the tail vein; therefore, it triggers the activation of circulating platelets without leading animals to death.

In experiments in nondiabetic wild-type and Dicer1^*Pf4Δ/Pf4Δ*^ mice aimed at assessing whether thrombin modifies Dicer1, miR-223-3p (micro RNA 223-3p), P2RY12 mRNA, and P2Y_12_ protein expressions in vivo, blood was collected 60 minutes after thrombin injection. In experiments in diabetic mice, blood was collected 60, 120, and 240 minutes after thrombin injection. These time points were selected based on the results of in vitro experiments showing that 60 minutes was the time point at which Dicer1 and microRNA-223 expressions were increased, and P2Y_12_ mRNA and protein expression were decreased. Blood was collected by cardiac puncture from mice under total anesthesia (tiletamine and zolazepam 50 mg/kg) and anticoagulated with sodium citrate 4% (1:10).^21^ Ultrapurified platelets were obtained by immunomagnetic cell sorting, as described.^22^

#### ADP-Triggered Platelet Pulmonary Microembolism

Mice were injected with human thrombin (500 U/kg) or with saline (control group) and after 60 minutes, a mixture of 2-methylthioadenosine 5′-diphosphate (20 mg/kg; Santa Cruz, sc-203464) and epinephrine (30 mg/kg; Mascia Brunelli) in 100 μL of PBS was administered into one of the tail veins.^23^ Time to cessation of respiration (time to the onset of respiratory arrest that lasted for at least 2–3 minutes) was recorded for a maximum of 30 minutes. Platelet count was performed before and 60 minutes after thrombin injection. Sixty minutes after 2-methylthioadenosine 5′-diphosphate injection, mice were euthanized by an overdose of anesthesia, the chest was opened, the trachea was cannulated, and the lungs were perfused with a fixing solution (10% buffered formalin; Bio Optica, Milan, Italy). The trachea was then ligated and removed together with the lungs, which were rinsed in cold saline and then fixed in 10% formalin for 24 hours. The right-lower lobe was embedded in paraffin, and several sections, 5- to 6-µm-thick, were cut and stained with hematoxylin and eosin to reveal platelet-rich thrombi. The specimens were examined under a light microscope by a pathologist unaware of the experimental groups. At least 10 fields, at a magnification of ×400, were observed for every specimen.

### Studies in Human Platelets

#### Blood Sampling, Platelet Purification, and Preparation

Venous blood was collected from healthy volunteers and patients with T2DM in acid-citrate-dextrose (1:6 vol/vol), and ultrapurified platelets were obtained by immunomagnetic cell sorting, as described.^22^ Leukocyte contamination (CD45+) was <0.005%, yielding a degree of purity sufficient to permit a reliable interpretation of results as of exclusive platelet origin (Figure S1A and S1B). Platelet purification did not affect platelet reactivity (Figure S2A).

Purified platelets were incubated with vehicle (M199) or bovine thrombin (0.1 U/mL) at room temperature under nonstirring conditions, and at selected time intervals (0–240 minutes), purified platelets were centrifuged at 11, 000*g* for 2 minutes to stop the reaction.^22^

In selected experiments, platelets were treated for 2 hours before stimulation with thrombin with mRNA-translation inhibitors, that is, puromycin 1 mM (Sigma-Aldrich, Saint Louis, MO), which causes the premature release of nascent polypeptide chains, or cycloheximide 1 mM (Sigma-Aldrich), which blocks translational elongation, or with 1 U/ml apyrase to remove ADP. Puromycin did not affect platelet reactivity (Figure S2B).

#### Protein Expression: Western blotting

Resting and activated platelets were lysed in protein extraction buffer (Tris, 40 mM; NaCl, 0.3M; EDTA, 1 mM; Na_3_VO_4_; NaF; pH, 7.4; with NP-40 and protease inhibitors) for 40 minutes on ice and centrifuged for 10 minutes at 10, 000*g*, and protein concentration was measured. Protein lysates were separated by SDS-PAGE electrophoresis and then electrotransferred onto a nitrocellulose membrane. For Dicer1 detection, a mouse anti-human monoclonal antibody was used (1:1000; AbCam, cat.n. ab14601) as primary and an anti-mouse HRP (horseradish peroxidase)–conjugated antibody (1:5000; Bio-Rad, cat.n. 5178-2504) as secondary antibody. In selected experiments, a different anti-human Dicer1 antibody was used (1:300; Santa Cruz, cat.n. sc-136981). The α-tubulin was used for normalization (Cell Signaling, cat. n. 2144). The specificity of the anti-Dicer1 antibody was confirmed using appropriate positive and negative controls^24^ (Figure S3). To exclude that changes in Dicer1 expression were the consequence of the binding of the antibody to a truncated Dicer1 product formed upon cleavage by calpain,^9^ selected experiments were performed by preincubating platelets with the calpain inhibitor calpeptin at a concentration of 100 μM for 15 minutes at room temperature. Calpeptin activity was shown by assessing its effect on platelet α_IIb_β_3_ (integrin α _IIb_β_3_) activation measured by the binding of PAC-1 (monoclonal antibody PAC-1; BD Biosciences, cat.n. 340507) to platelets, given that calpain is involved in α_IIb_β_3_ activation.^25^ An isotypic antibody was used as a negative control (IgM Mouse-FITC; Beckman Coulter, cat.n. IM1269U).

P2Y_12_ expression was assessed using a rabbit monoclonal anti-P2Y_12_ antibody^8^ (Alomone Labs, Jerusalem, Israel; cat.n. APR-012; 1:500) as primary and an anti-rabbit HRP-conjugated (1:3000; Bio-Rad, cat.n. 5196-2504) as secondary antibody. Immunoreactivity was detected by chemiluminescence using ECL Western blotting substrate (Bio-Rad). The α-tubulin was used for normalization.

To exclude that changes in P2Y_12_ expression were the consequence of the binding of the antibody to a cleavage product of P2Y_12_ by thrombin, selected experiments were performed by incubating a platelet protein lysate with thrombin (0.1 U/mL) for 60 minutes and assessing P2Y_12_ expression as described above. As a positive control for thrombin cleavage activity, we assessed the cleavage of the N terminus of PAR1 (protease-activated receptor 1) by Western blotting with anti–N-terminus PAR1 antibody (Novus Biologicals; cat.n. NBP1-71770; 1:1000) raised against the soluble amino-terminal thrombin cleavage peptide region of PAR1 as primary and an anti-rabbit HRP-conjugated antibody (1:3000; Bio-Rad) as secondary antibody.

Densitometric analysis and quantification were performed using the ImageJ software (National Institutes of Health, United States).

### Dicer1 Expression by Liquid Chromatography-Tandem Mass Spectrometry Analysis

The liquid chromatography-tandem mass spectrometry analysis was performed by the TIGEM proteomics facility (Pozzuoli, Italy) using a NanoLC 1200 coupled via a nano-electrospray ionization source to the quadrupole-based Q Exactive HF benchtop mass spectrometer. All acquired raw files were processed using the Spectronaut software (18.0 license). Spectra were correlated with the Uniprot Human databases (version 2023) for protein assignment. Searches were performed with tryptic specifications and default settings for mass tolerances for both mass spectrometry and tandem mass spectrometry spectra. The Perseus software (version 1.6.2.3) was used to logarithmize, group, and filter the protein abundance. Further details are available in the Supplemental Material.

### Protein Synthesis: Radiolabeling With ^35^S-Methionine

Leukocyte-depleted platelets were suspended for 1 hour at 37 °C in M199 without methionine. ^35^S-methionine (50 μCi) was then added, and after 15 minutes, platelets were stimulated with thrombin (0.1 U/mL) for increasing time intervals (0–240 minutes). Platelets were then centrifuged (20, 000 g, 5 minutes), and the cell pellets were collected, resuspended in medium, and lysed by freezing and thawing. Lysates were placed in a Dicer1-specific ELISA plate for 2 hours (Cloud-Clone Corp, Houston, TX), and the wells were washed 5× with PBS to remove unbound material. Proteins bound to the plates were subsequently detached using a triethanolamine buffer solution and TRIS of 1 M and pH 8.2 for 10 minutes, suspended in liquid scintillation fluid, and counted in a scintillation counter (1900 TR liquid scintillation analyzer, PACKARD).^22^

### Protein Synthesis: mTOR and Mnk1 Phosphorylation

Protein lysates from resting and thrombin-activated platelets (0.1 U/mL for 5 and 60 minutes) were separated by SDS-PAGE. mTOR and Mnk1 phosphorylations were assessed by incubating the membranes with a rabbit anti-human phospho-mTOR antibody or a rabbit anti-human phospho-Mnk1 antibody (1:1000; both from Cell Signaling, cat. n. 2971 and 2111). Total mTOR and Mnk1 were used for normalization (1:1000; both from Cell Signaling, cat. n. 2972 and 2195).

### mRNA and microRNA Expressions

The mRNA expression was measured by reverse transcription quantitative polymerase chain reaction (PCR) using the Brilliant SYBR Green QPCR Master Mix 2x with the Mx3000P qPCR System (Stratagene, Santa Clara, CA). Real-time PCR results were analyzed and presented using the 2^-ΔΔCt^ method, with B2M (beta-2-microglobulin) and GAPDH serving as housekeeping genes for normalization.

The microRNA expression was assessed using the miRCURY LNA Universal RT microRNA PCR system (Exiqon, Vedbaek, Denmark). Absolute miR-223-3p quantification was obtained using a 6-point standard curve prepared using a synthetic RNA oligonucleotide corresponding to the sequence of miR-223-3p. An exogenous reference RNA, UniSp6 (Exiqon), was used as a housekeeping control to normalize the amount of pre-miR-223.

Primer sequences and PCR product lengths are shown in Table S1.

### In Vitro Dicer-Activity Assay

Ultrapurified platelets, resting or stimulated with thrombin (0.1 U/mL) for 5 minutes, were lysed with protein extraction buffer, and 500 μg of proteins were incubated overnight with an anti-Dicer1 antibody (5 μg) and protein-G agarose beads (Sigma-Aldrich) under continuous rotation at 4 °C to immunoprecipitate and remove Dicer1 from the platelet lysate. The platelet lysate was then concentrated using Amicon Ultra-0.5 centrifugal filters and incubated for 1 h at 37 °C with 20 ng of a platelet microRNA/pre-microRNA extract obtained with the mirVana microRNA isolation kit, to measure miR-223-3p, or with a total RNA extract to assess P2RY12 mRNA expression, resuspended in a reaction buffer containing 20-mmol/L TrisHCl, pH 7.5, 5-mmol/L MgCl_2_, 1-mmol/L dithiothreitol, 1-mmol/L ATP, and 5% Superase In (Life Technologies).^26^ RNA was then extracted, and microRNA-223 and P2RY12 mRNA expression were assessed by real-time PCR, as described above.

### Statistics

Results are shown as dot plots showing mean and SD. Normality was assessed for each data set using the D’Agostino-Pearson Omnibus Test. For sample sizes <10, a nonparametric test was applied, independently of data distribution. A Mann-Whitney *U* test was used to analyze differences between 2 groups. For ≥3 unmatched groups, differences were analyzed using 1-way ANOVA, followed by the Bonferroni multiple comparisons test for normally distributed data or the Kruskal-Wallis test or the Friedman test followed by the Dunn multiple comparisons test for nonnormally distributed data. For comparisons involving 2 independent variables, changes within each group were assessed using the Friedman test followed by the Dunn multiple comparisons test or the Kruskal-Wallis test, as appropriate. Comparisons between the 2 groups at each time point were performed using the Mann-Whitney *U* test, with Bonferroni correction for multiple comparisons. A *P* value of <0.05 was considered statistically significant. Correlations were analyzed with the Pearson correlation coefficient as the data followed a normal distribution. The normality of residuals was assessed using the D’Agostino and Pearson test (for n≥8) or the Shapiro-Wilk test (for n<8). All the statistical analyses were performed using the GraphPad PRISM 8 software, version 8.3.1, on macOS.

Detailed description of liquid chromatography-tandem mass spectrometry analysis, extraction of platelet cytoskeleton, flow cytometry, RNA and microRNA extraction and real-time PCR, murine megakaryocytes culture and transfection, isolation of platelet microparticles (PMPs), immunofluorescence and confocal microscopy, measurement of cAMP, and vasodilator-stimulated phosphoprotein (VASP) phosphorylation assay are presented in the Supplemental Methods.

Representative images were selected based on their clarity and to best illustrate the observed effects reflecting the mean values of the experimental repetitions.

Please see the Major Resources Table in the Supplemental Material.

## Results

### Thrombin Induces Dicer1 Neosynthesis in Platelets, a Process Dysregulated in Patients With T2DM

**Figure 1.**
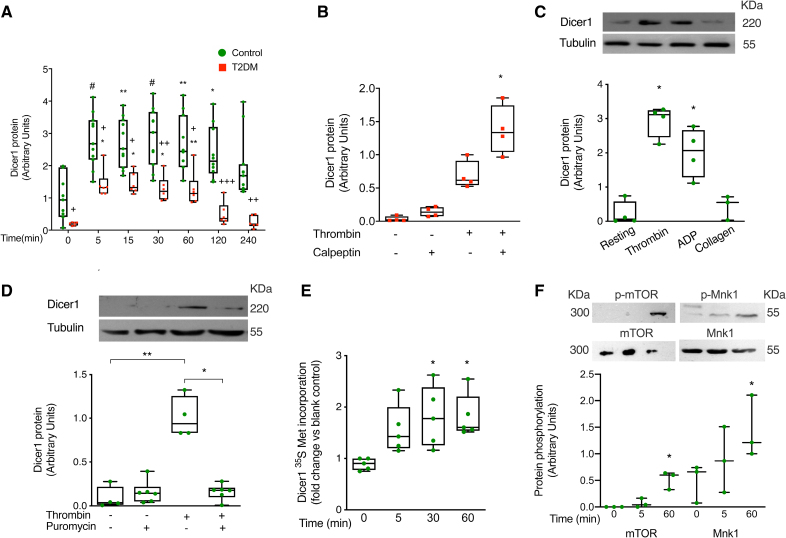
**Dicer1 (ribonuclease III enzyme Dicer-1) is neosynthesized by activated platelets, a process altered in type 2 diabetes (T2DM) platelets. A**, Western blotting of platelet expression of Dicer1 upon stimulation with thrombin (0.1 U/mL). α-tubulin was used as a loading control. Optical densitometric analysis was performed using the Image J software, and results are expressed in arbitrary units. Results are shown as mean with SD showing individual points; n=11 biological replicates for controls and n=6 biological replicates for T2DM. For controls: #*P*=6.5×10^−4^ time 5 vs time 0, **P=0.0012 time 15 vs time 0, #*P*=3.2×10^−4^ time 30 vs time 0, **P=0.0013 time 60 vs time 0, *P=0.0327 time 120 vs time 0, and *P*=0.8378 time 240 vs time 0. The Kruskal-Wallis test followed by the Dunn multiple comparisons test. For T2DM: *P=0.0197 time 5 vs time 0, *P=0.0451 time 15 vs time 0, *P=0.0451 time 30 vs time 0, **P=0.0030 time 60 vs time 0, and *P*>0.9999 times 120 and 240 vs time 0. The Friedman test was followed by the Dunn multiple comparisons test. Comparison between controls and T2DM: +*P*=0.0405 controls vs T2DM at time 0, +*P*=0.0139 controls vs T2DM at time 5, +*P*=0.0113 controls vs T2DM at time 15, ++*P*=0.0065 controls vs T2DM at time 30, +*P*=0.0168 controls vs T2DM at time 60, +++*P*=8×10^−4^ controls vs T2DM at time 120, and ++*P*=0.0052 controls vs T2DM at time 240. The Mann-Whitney *U* test, with Bonferroni correction for multiple comparisons. Note that, as shown in Figure S4 and Figure 1C, Dicer1 protein can be detected in resting platelets. Differences in the expression of Dicer1 in resting platelets are linked to the exposure time of the Western blotting films to chemiluminescence, which was different in every experiment to obtain informative pictures. Comparable results were obtained using a different anti-Dicer1 antibody (Figure S19). **B**, Western blotting of Dicer1 protein expression upon T2DM platelet stimulation with thrombin (0.1 U/mL) for 120 minutes, after pretreatment with calpeptin 10 μM or its vehicle for 15 minutes. The β-actin was used as a loading control. Optical densitometric analysis was performed using the Image J software, and results are expressed in arbitrary units. Results are shown as mean with SD showing individual points; n=4 biological replicates; *P=0.0427 treated with thrombin vs treated with calpeptin and thrombin; the Friedman test was followed by the Dunn multiple comparisons test. **C**, Western blotting of platelet expression of Dicer1 upon stimulation for 60 minutes with different agonists (thrombin, 0.1 U/mL; ADP, 10 μM; and collagen, 2 μg/mL). α-tubulin was used as a loading control. Optical densitometric analysis was performed using the Image J software, and results are expressed in arbitrary units. Results are shown as mean with SD showing individual points; n=4 biological replicates for resting, thrombin, and ADP; and n=3 biological replicates for collagen. *P=0.0279 resting vs thrombin; *P=0.0440 resting vs ADP; and *P*=0.8491 resting vs collagen; the Kruskal-Wallis test followed by the Dunn multiple comparisons test. **D**, Effect of preincubation with puromycin, or its vehicle, on thrombin (0.1 U/mL)-induced synthesis of Dicer1 in platelets. α-tubulin was used as a loading control. Optical densitometric analysis was performed using the Image J software, and results are expressed in arbitrary units. Results are shown as mean with SD showing individual points, for resting, for thrombin n=4, for resting+puromycin, and for thrombin+puromycin n=6 biological replicates. **P=6.9×10^−3^ resting vs thrombin; *P=0.0340 thrombin+puromycin vs thrombin; and *P*>0.99 resting vs resting+puromycin; the Kruskal-Wallis test followed by the Dunn multiple comparisons test. **E**, Incorporation of ^35^S-methionine into Dicer1 protein over time in thrombin (0.1 U/mL)-stimulated platelets. ^35^S-methionine incorporation is expressed as fold increase over baseline. Results are shown as mean with SD showing individual points; n=5 biological replicates. *P=0.0258 time 0 vs time 30, *P=0.0153 time 0 vs time 60, and *P*=0.1707 time 0 vs time 5; the Kruskal-Wallis test followed by the Dunn multiple comparisons test. Individual points are fold changes of the radioactive signal relative to the signal obtained in samples at time 0 with medium without ^35^S-methionine, which was used as the blank control. **F**, Western blotting of phosphorylation of mTOR (mammalian target of rapamycin) and Mnk1 (mitogen-activated protein kinase-interacting kinase 1) in resting platelets and in platelets upon stimulation for 5 or 60 minutes with thrombin. Total mTOR and Mnk1 were used as loading controls. Optical densitometric analysis was performed using the Image J software, and results are expressed in arbitrary units. Results are shown as mean with SD showing individual points; n=3 biological replicates. For p-mTOR: *P*>0.99 time 0 vs time 5, *P=0.0394 time 0 vs time 60; for p-Mnk1 (phospho-mitogen-activated protein kinase-interacting serine/threonine-protein kinase 1) *P*=0.4413 time 0 vs time 5, and *P=0.0286 time 0 vs time 60; the Friedman test was followed by the Dunn multiple comparisons test.

Dicer1 increased significantly in platelets upon thrombin stimulation. The increase was about 2-fold and rapid, starting already 3 minutes after stimulation (Figure S4) and maximal after 5 minutes, and it persisted for up to 120 minutes in healthy controls (Figure 1A). Dicer1 expression was markedly decreased in resting platelets from patients with T2DM compared with controls, confirming previous results.^9^ Upon thrombin stimulation, although a similar early increase in Dicer1 was observed, Dicer1 levels declined more rapidly than in healthy controls, returning to baseline by 60 minutes (Figure 1A). The incubation of T2DM platelets with the calpain inhibitor calpeptin^9^ increased Dicer1 levels, as expected (Figure 1B).

Dicer1 was detected in resting, ultrapurified human platelets by Western blotting (Figure S5A), flow cytometry (Figure S5B), immunofluorescence (Figure S5C), and confocal microscopy (Figure S5D). It was localized in the platelet cytoplasm under resting conditions and bound to the cytoskeleton after stimulation with thrombin (Figure S5E). We observed rapid Dicer1 increase after thrombin stimulation also by flow cytometry (Figure S6). An increase in Dicer1 was also observed after ADP stimulation, but no statistically significant difference was observed after collagen stimulation (Figure 1C). Dicer1 increase induced by thrombin was not affected by preincubation with the calpain inhibitor calpeptin (Figure S7A and S7B), thus excluding that changes in Dicer1 expression were the consequence of the binding of the antibody to a Dicer1 cleavage-product formed by calpain^9^ and also that the platelet isolation procedure resulted in calpain activation. Moreover, Dicer1 increase induced by thrombin was still evident when platelets were preincubated with apyrase, suggesting that Dicer synthesis is not strictly dependent on ADP stimulation (Figure S7C).

The increase in Dicer1 induced by thrombin was no longer statistically significant when platelets were preincubated with 2 protein synthesis inhibitors, either puromycin (Figure 1D; Figure S8A) or cycloheximide (Figure S8B).

To confirm de novo synthesis of Dicer1, we incubated platelets with ^35^S-methionine and measured its incorporation into new protein. Radiolabeled methionine was readily incorporated into Dicer1 in thrombin-activated platelets in a time-dependent way (Figure 1E).

To definitively prove the identity of Dicer1 as the protein neosynthesized upon thrombin stimulation, we performed liquid chromatography-tandem mass spectrometry analysis. Dicer was detected in resting platelets and increased upon stimulation with thrombin (Figure S9).

Platelet stimulation with thrombin activated mTOR and Mnk1 after 60 minutes; some activation of Mnk1 was evident already after 5 minutes, but it was not statistically significant (Figure 1F).

### Dicer1 mRNA in Platelets Is Not Modified by Thrombin Stimulation

**Figure 2.**
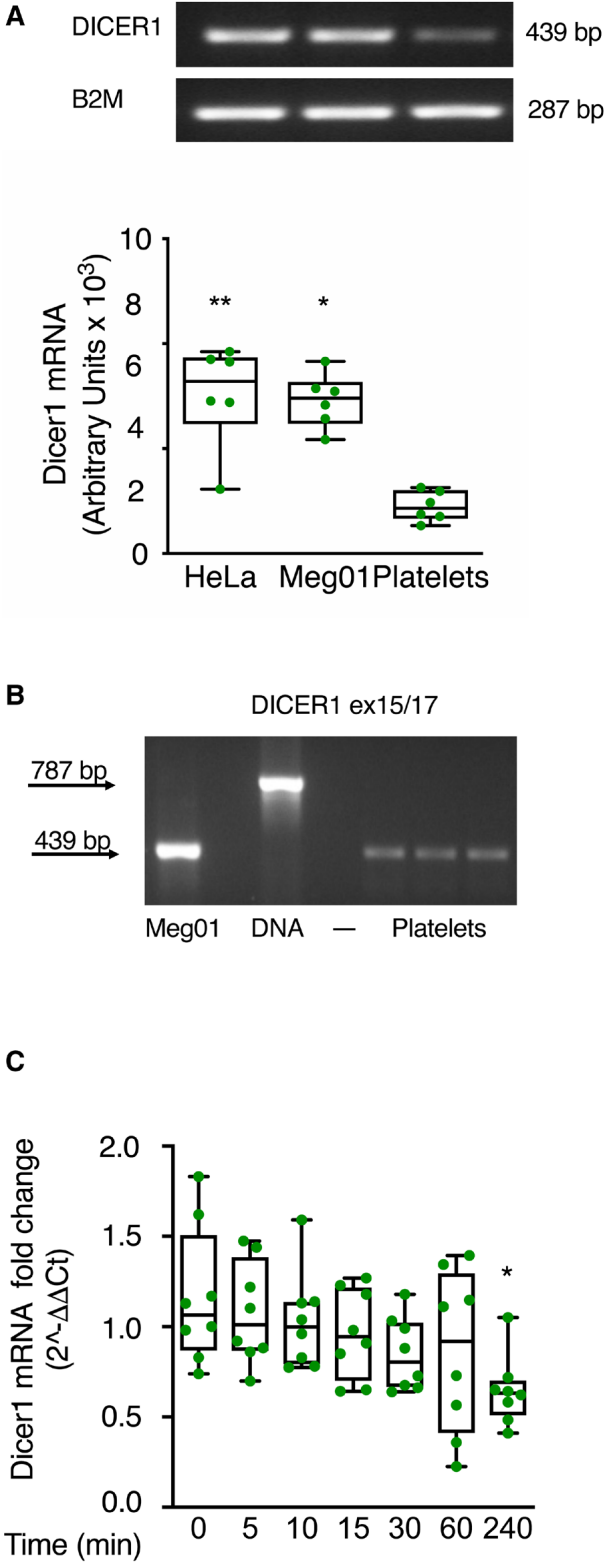
**Platelets contain mRNA for Dicer1 (ribonuclease III enzyme Dicer-1). A**, Polymerase chain reaction (PCR) of Dicer1-coding mRNA in platelets, and in MEG-01 (MEG-01 cell line) and HeLa (HeLa cell line) cells as a comparison. Optical densitometric analysis was performed using the Image J software, and results are expressed in arbitrary units normalized to B2M (beta-2-microglobulin), which was used as housekeeping, and are shown as mean with SD showing individual points; n=6 biological replicates. **P=0.0061 HeLa vs platelets and *P=0.0283 MEG-01 vs platelets; the Kruskal-Wallis test followed by the Dunn multiple comparisons test. **B**, PCR of Dicer1-coding mRNA. Dicer1 mRNA in platelets was amplified by using primers designed to identify both premRNA and mature mRNA. Dicer1 mRNA is present in platelets in its mature form (439 bp). MEG-01 RNA and peripheral blood mononuclear cell (PBMC) DNA served as controls for primer efficiency. **C**, Real-time PCR of Dicer1-coding mRNA over time after platelet stimulation with thrombin (0.1 U/mL). Results are presented as 2^-ΔΔCt^, with B2M and GAPDH serving as housekeeping genes for normalization. Results are shown as mean with SD showing individual points; n=8 biological replicates. **P=0.0085 time 240 vs time 0; *P*>0.99 times 5, 10, and 15 vs time 0; *P*=0.5417 time 30 vs time 0; and *P*=0.9238 time 60 vs time 0; the Kruskal-Wallis test followed by the Dunn multiple comparisons test. A decrease in Dicer1 mRNA over time was also observed in unstimulated platelets (data not shown). NTC indicates no template control.

mRNA for Dicer1 was found in platelets, although in lower amounts compared with HeLa (HeLa cell line) and MEG-01 (MEG-01 cell line) cells used as positive controls (Figure 2A). Mature Dicer1 mRNA (439 bp), but not its premRNA (precursor messenger RNA; 787 bp), was detected in resting platelets by PCR (Figure 2B). Stimulation with thrombin did not modify Dicer1 mRNA levels for up to 60 minutes, while a slight reduction was observed at 4 hours (Figure 2C). Dicer1 mRNA reduction at 4 hours was not due to its release from platelets upon activation (Figure S10A).

### Dicer1-Driven Modulation of Platelet microRNA Expression Is Altered in T2DM Platelets

**Figure 3.**
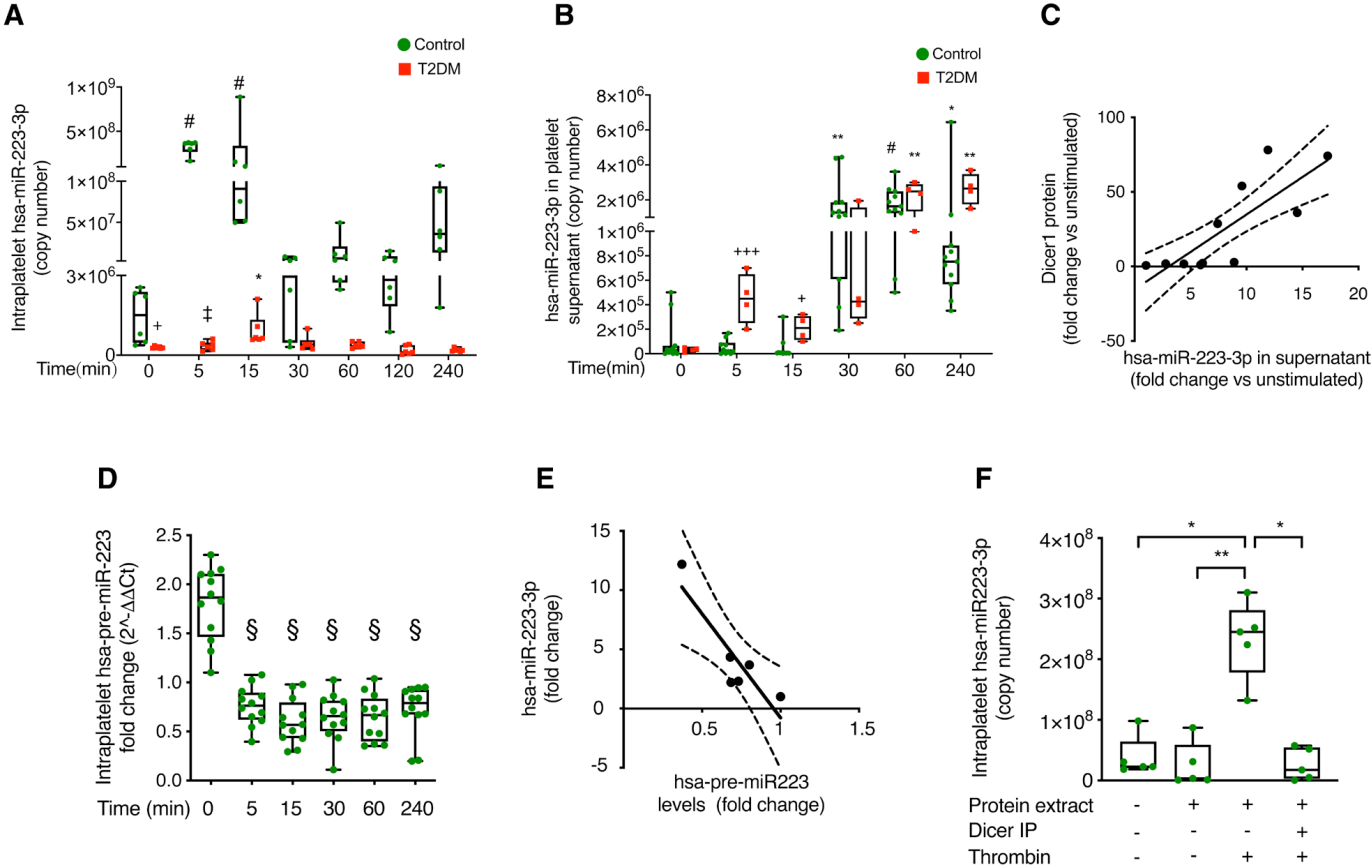
**Thrombin-induced increase in Dicer1 (ribonuclease III enzyme Dicer-1) modifies miR-223-3p (micro RNA 233-3p) levels in platelets, a process altered in type 2 diabetes (T2DM) platelets. A**, Absolute quantification of intraplatelet mature hsa-miR-223-3p (homo sapiens micro RNA 223-3p) over time upon stimulation with thrombin (0.1 U/mL), as measured by real-time polymerase chain reaction (PCR). The expression of hsa-miR-223-3p is reported as copy number. Results are shown as mean with SD showing individual points; n=12 biological replicates for controls and n=6 biological replicates for T2DM. For controls: #*P*=1×10^−4^ time 5 vs time 0, #*P*=9×10^−4^ time 15 vs time 0, *P*=0.7311 time 30 vs time 0, *P*=0.8579 time 60 vs time 0, and *P*>0.99 time 240 vs time 0; the Kruskal-Wallis test followed by the Dunn multiple comparisons test. For T2DM: *P=0.0197 time 15 vs time 0 and *P*>0.9999 for all the other comparisons. The Friedman test was followed by the Dunn multiple comparisons test. Comparison between controls and T2DM: +*P*=0.0172 controls vs T2DM at time 0, ‡*P*=1.6×10^−5^ controls vs T2DM at time 5, *P*=0.9273 controls vs T2DM at time 15, *P*=0.2767 controls vs T2DM at time 30, *P*=0.8341 controls vs T2DM at time 60, *P*=0.3197 controls vs T2DM at time 120, and *P*=0.1379 controls vs T2DM at time 240. The Mann-Whitney *U* test, with Bonferroni correction for multiple comparisons. **B**, Absolute quantification of mature hsa-miR-223-3p in the platelet supernatant over time upon stimulation with thrombin (0.1 U/mL) as measured by real-time PCR. The expression of hsa-miR-223-3p is reported as a copy number. Results are shown as mean with SD showing individual points; n=12 biological replicates for controls and n=4 biological replicates for T2DM. For controls: **P=0.0011 time 30 vs time 0, #*P*=1×10^−4^ time 60 vs time 0, *P=0.0316 time 240 vs time 0, and *P*>0.9999 time 5 and time 15 vs time 0; the Kruskal-Wallis test followed by the Dunn multiple comparisons test. For T2DM: **P=0.0065 time 60 vs time 0, **P=0.0016 time 240 vs time 0, *P*=0.1881 time 5 vs time 0, *P*>0.9999 time 15 vs time 0, and *P*=0.6528 time 30 vs time 0. The Friedman test was followed by the Dunn multiple comparisons test. Comparison between controls and T2DM: +++*P*=5.2×10^−4^ controls vs T2DM at time 5, +*P*=0.0489 controls vs T2DM at time 15, *P*=0.9481 controls vs T2DM at time 240, and *P*>0.9999 for all the other comparisons. The Mann-Whitney *U* test, with Bonferroni correction for multiple comparisons. **C**, Correlation between the change in the expression of Dicer1 in healthy control platelets by western blotting and released hsa-miR-223-3p by real-time PCR upon thrombin stimulation for 60 minutes (Pearson r=0.81; R^2^=0.65; n=11; *P*=0.0027). Changes in expression are reported as fold change with respect to unstimulated. **D**, Expression of hsa-pre-miR-223 in platelets over time upon stimulation with thrombin (0.1 U/mL) as measured by real-time PCR. Results are presented as 2^-ΔΔCt^, and the expression of hsa-pre-miR-223 is normalized to the exogenous UniSp6 RNA. Results are shown as mean with SD showing individual points; n=12 biological replicates. §*P*=7×10^−7^ time 5 vs time 0, §*P*=1×10^−7^ time 15 vs time 0, §*P*=1×10^−7^ time 30 vs time 0, §*P*=2×10^−7^ time 60 vs time 0, and §*P*=4×10^−7^ time 240 vs time 0; 1-way ANOVA test followed by the Dunnett multiple comparisons test. **E**, Correlation between the change in expression of hsa-pre-miR-223 and mature hsa-miR-223-3p in healthy control platelet supernatants upon thrombin stimulation for 60 minutes (Pearson r=0.88; R^2^=0.78; n=10; *P*=0.0007). Changes in expression are reported as fold change with respect to unstimulated. **F**, In vitro Dicer-activity assay. A protein extract of thrombin-stimulated (5 minutes), but not of resting healthy control platelets, caused the maturation of an exogenously added platelet pre-miR-223, while the same extract depleted of Dicer1 by immunoprecipitation did not induce miR-223 maturation. Results are shown as mean with SD showing individual points; n=5 biological replicates. *P=0.0432 first group vs third group, **P=0.0082 second group vs third group, and *P=0.0138 fourth group vs third group; the Kruskal-Wallis test followed by the Dunn multiple comparisons test.

To test whether Dicer1 neosynthesis triggers pre-microRNA processing, we measured pre-miR-223-3p and mature miR-223-3p, one of the most abundant microRNAs, in extracts and supernatants of thrombin-stimulated platelets. In healthy subjects, intraplatelet miR-223-3p increased significantly starting from 5 minutes after stimulation and reaching a maximum at 15 minutes and then decreased starting from the 30th minute (Figure 3A), that is, the time at which a significant release of miR-223-3p in the supernatant was observed. Indeed, thrombin triggered the release of a large number of mature miR-223-3p copies in the platelet supernatant, which started 30 minutes after stimulation and was maximal at 60 minutes (Figure 3B). In patients with T2DM, the miR-223-3p expression was markedly decreased in resting platelets compared with controls, confirming previous results.^9^ Thrombin stimulation also induced an early increase in miR-223-3p, but this was transient and returned to baseline already by 30 minutes (Figure 3A). Accordingly, release of miR-223-3p in the supernatant of T2DM platelets was significantly delayed, becoming evident only at 60 minutes post-stimulation (Figure 3B). Upon treatment with calpeptin, the maturation of miR-223-3p persisted, similar to controls (Figure S11).

Maturation of miR-223-3p induced by thrombin was no longer statistically significant in healthy platelets preincubated with the protein synthesis inhibitor cycloheximide (Figure S12). Moreover, a significant, strong direct correlation was found between thrombin-induced Dicer1 neosynthesis and the increase in mature miR-223-3p (Figure 3C).

Inversely, intraplatelet pre-miR-223 decreased after thrombin stimulation, with a maximal reduction between 15 and 60 minutes (Figure 3D), a reduction not due to its release from platelets upon activation (Figure S10B). A significant and strong inverse correlation was found between thrombin-induced pre-microRNA decrease and mature miR-223-3p increase (Figure 3E). To conclusively show that neosynthesis of Dicer1, and not of another protein, was responsible for the observed miR-223-3p increase, we performed an in vitro Dicer-activity assay. A protein extract of thrombin-stimulated, but not of resting, platelets caused the maturation of miR-223-3p from an exogenously added platelet pre-microRNA/microRNA extract; on the contrary, no statistically significant difference was observed in miR-223-3p maturation following immunoprecipitation of Dicer1 (Figure 3F; Figure S13).

Finally, as a control, we measured miR-451a in platelets, a microRNA the processing of which is independent of Dicer1^27^; no statistically significant difference in miR-451a expression was observed after stimulation with thrombin (Figure S14A and S14B), indirectly confirming that thrombin-induced miR-223-3p maturation in platelets is the consequence of Dicer1 neosynthesis.

### Dicer1-Dependent Modulation of Platelet mRNA and Protein Expression Is Dysregulated in T2DM

**Figure 4.**
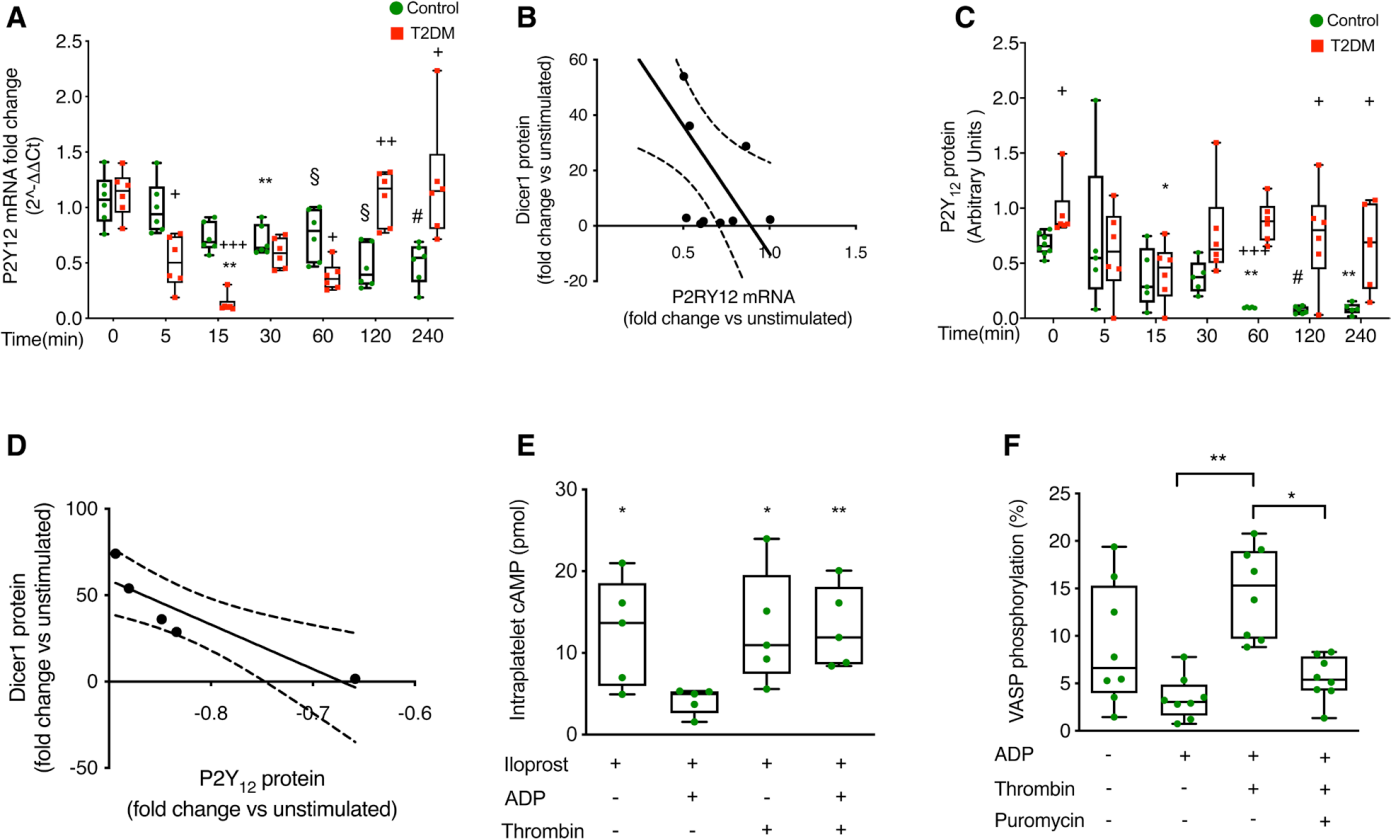
**Thrombin-induced increase in Dicer1 (ribonuclease III enzyme Dicer-1) reduces P2Y12 expression and function in platelets, a process altered in type 2 diabetes (T2DM) platelets. A**, Expression of P2Y_12_ (purinergic receptor P2Y_12_) coding mRNA over time upon platelet stimulation with thrombin (0.1 U/mL), as measured by real-time polymerase chain reaction (PCR). Results are presented as 2^-ΔΔCt^, with B2M (beta-2-microglobulin) and GAPDH serving as housekeeping genes for normalization. Results are shown as mean with SD showing individual points; n=12 biological replicates for controls and n=6 biological replicates for T2DM. For controls: **P=0.0022 time 30 vs time 0, §*P*=1×10^−6^ time 60 vs time 0, §*P*=1×10^−6^ time 120 vs time 0, #*P*=6×10^−4^ time 240 vs time 0, *P*>0.99 time 5 vs time 0, and *P*=0.0754 time 15 vs time 0; 1-way ANOVA test followed by the Dunnett multiple comparisons test. For T2DM: **P=0.0064 time 0 vs time 15, *P*=0.4851 time 0 vs time 5, *P*=0.1580 time 0 vs time 60, and *P*>0.9999 for all the other time points vs time 0. The Friedman test was followed by the Dunn multiple comparisons test. Comparison between controls and T2DM: +*P*=0.03245 controls vs T2DM at time 5, +++*P*=2.3×10^−5^ controls vs T2DM at time 15, +*P*=0.0409 controls vs T2DM at time 60, ++*P*=0.0043 controls vs T2DM at time 120, +*P*=0.0472 controls vs T2DM at time 240, and *P*>0.9999 controls vs T2DM at time 0 and time 30. The Mann-Whitney *U* test, with Bonferroni correction for multiple comparisons. **B**, Correlation between the change in expression of Dicer1 protein and P2Y_12_-coding mRNA upon thrombin stimulation for 60 minutes (Pearson r=−0.63; R^2^=0.39; n=11; *P*=0.0205). **C**, Western blotting of P2Y_12_ protein over time upon platelet stimulation with thrombin (0.1 U/mL). Tubulin was used as a loading control. Optical densitometric analysis was performed using Image J software, and results are expressed in arbitrary units. Results are shown as mean with SD showing individual points. For controls: n=8 biological replicates for time 0; n=5 biological replicates for times 5, 15, 30, 60, and 240; and n=6 biological replicates for time 120. For T2DM: n=6 biological replicates. For controls: **P=0.0074 time 60 vs time 0, #*P*=4.1×10^−4^ time 120 vs time 0, **P=0.0021 time 240 vs time 0, *P*>0.9999 time 5 vs time 0, *P*=0.4767 time 15 vs time 0, and *P*=0.8888 time 30 vs time 0; the Kruskal-Wallis test followed by the Dunn multiple comparisons test. For T2DM: *P=0.0254 time 15 vs time 0, *P*=0.5279 time 5 vs time 0, *P*=0.7223 time 30 vs time 0, *P*=0.7923 time 240 vs time 0, and *P*>0.9999 time 60 and time 120 vs time 0. The Friedman test was followed by the Dunn multiple comparisons test. Comparison between controls and T2DM: +*P*=0.0471 controls vs T2DM at time 0, +++*P*=4.5×10^−5^ controls vs T2DM at time 60, +*P*=0.0283 controls vs T2DM at time 120, +*P*=0.0471 controls vs T2DM at time 240, *P*=0.5657 controls vs T2DM at time 30, and *P*>0.9999 controls vs T2DM at time 5 and time 15. The Mann-Whitney *U* test, with Bonferroni correction for multiple comparisons. **D**, Correlation between the change in expression of platelet Dicer1 protein and P2Y_12_ protein upon thrombin stimulation for 60 minutes (Pearson r=−0.89; R^2^=0.80; n=5; *P*=0.0380). **E**, cAMP levels in platelets preincubated or not with thrombin. Iloprost increased cAMP levels in resting platelets, an effect blunted by stimulation with ADP. Iloprost was still able to raise cAMP levels in platelets preincubated with thrombin, but, in this case, stimulation with ADP was no longer able to reduce cAMP. Results are shown as mean with SD showing individual points; n=5 biological replicates. *P=0.0247 second group vs first group, *P=0.0119 second group vs third group, **P=0.0088 second group vs fourth group, and *P*=0.9148 third group vs fourth group; the Kruskal-Wallis test followed by the Dunn multiple comparisons test. **F**, Vasodilator-stimulated phosphoprotein (VASP) phosphorylation induced by PGE_1_. Phosphorylation of VASP induced by PGE_1_ (prostaglandin E_1_) in resting platelets (first group) was blunted by the stimulation with ADP (second group). Following thrombin activation, ADP was no longer able to reduce PGE_1_-induced VASP phosphorylation (third group). Incubation of platelets with puromycin before stimulation with thrombin restored the ability of ADP to reduce PGE_1_-induced VASP phosphorylation (fourth group). Results are shown as mean with SD showing individual points; n=8 biological replicates. **P=0.0019 second group vs third group, *P=0.0338 fourth group vs third group, and *P*>0.99 first group vs third group; the Kruskal-Wallis test followed by the Dunn multiple comparisons test.

We then investigated whether thrombin-triggered miR-223-3p maturation affects target mRNAs in platelets. Given that one of the main targets of miR-223-3p in platelets is P2RY12 mRNA,^28^ we measured the levels of the latter in resting and thrombin-stimulated platelets. In healthy subjects, thrombin induced a time-dependent downregulation of platelet P2RY12 mRNA that reached significance at 30 minutes and persisted for 4 hours. In patients with T2DM, thrombin also triggered a rapid reduction of P2RY12 mRNA levels in platelets as early as 15 minutes post-stimulation, but, in contrast to healthy platelets, this effect was transient, with mRNA levels returning to baseline by 120 minutes (Figure 4A). P2RY12 mRNA reduction was not due to its release from platelets upon activation; in fact, P2RY12 mRNA was detected in platelet supernatants only after 240 minutes of stimulation with thrombin in 2 of 4 samples and only after 31 PCR cycles (Figure S10C). Moreover, an inverse correlation between the increase in intraplatelet Dicer1 and the decrease in P2RY12 mRNA induced by thrombin was evident (Figure 4B).

To conclusively show that neosynthesis of Dicer1, and not of another protein, was responsible for the observed P2RY12 mRNA decrease, we performed an in vitro Dicer-activity assay. A protein extract of thrombin-stimulated, but not of resting, platelets caused the decrease in P2RY12 mRNA from an exogenously added platelet RNA extract^1,26^; on the contrary, using the same extract depleted of Dicer1 by immunoprecipitation, no statistically significant difference was observed regarding P2RY12 mRNA (Figure S15).

We then investigated platelet P2Y_12_ expression following thrombin stimulation. In healthy subjects, platelet P2Y_12_ levels significantly decreased after 60 minutes and remained suppressed for up to 240 minutes. In patients with T2DM, the baseline platelet P2Y_12_ protein expression was increased, confirming previous results,^8^ while thrombin induced a more rapid downregulation of P2Y_12_ protein, already evident at 15 minutes, which, differently from healthy platelets, returned to baseline at 120 minutes (Figure 4C). A significant inverse correlation was found between the increase in Dicer1 and the decrease in P2Y_12_ triggered by thrombin (Figure 4D). To exclude that thrombin, that is, a protease, could cleave P2Y_12_ in this way decreasing P2Y_12_ expression, we incubated a platelet protein extract with thrombin. Upon 60 minutes of incubation, no difference was observed regarding the expression of P2Y_12_, while that of PAR1, the platelet receptor cleaved by thrombin, decreased (Figure S16).

To clarify whether Dicer1-induced P2Y_12_ decrease has a functional impact on platelets, we measured P2Y_12_-mediated downregulation of adenylylcyclase, a major downstream effect of P2Y_12_ stimulation.^29^ The stable prostacyclin analog iloprost, an adenylylcyclase activator, increased platelet cAMP, an effect blunted by ADP.^29^ Iloprost was still able to raise cAMP in platelets preincubated with thrombin, but, in this case, stimulation with ADP was no longer able to reduce cAMP (Figure 4E). These data were confirmed by the measurement of VASP phosphorylation. VASP, a member of a conserved family of actin regulatory proteins, is dephosphorylated in platelets by ADP via P2Y_12_-mediated Gαi (subunit α of the inhibitory G protein) activation.^30^ PGE_1_ (postaglandin E_1_)-induced VASP phosphorylation in platelets, which was inhibited by stimulation with ADP.^30^ Following preincubation with thrombin, ADP no longer reduced PGE_1_-induced VASP phosphorylation. Treatment of platelets with the mRNA-translation inhibitor puromycin before stimulation with thrombin restored the ability of ADP to blunt PGE_1_-induced VASP phosphorylation (Figure 4F).

### Release of miR-223-3p but Not of Dicer1 Into PMPs

The delivery of Ago2 (argonaute 2) from platelets to endothelial cells via PMPs has been reported.^31^ Therefore, we assessed whether Dicer1 is also released into PMPs. Stimulation of platelet-rich plasma with A23187, TRAP (thrombin receptor activating peptide), collagen, or TRAP+collagen induced the generation of PMPs, with A23187 being the most effective stimulus (Figure S17A and S17B). Western blotting of PMP lysates, independently from the stimulus, did not allow to detect Dicer1, while it confirmed the presence of Ago2 (Figure S17C). However, PMPs contained miR-223-3p, which increased after stimulation with thrombin, an effect inhibited by preincubation with puromycin (Figure S17D), showing that the passage of microRNA-223 into PMPs is dependent on protein neosynthesis.

### Thrombin Induces Dicer1 Neosynthesis and Regulates Platelet Gene Expression In Vivo

**Figure 5.**
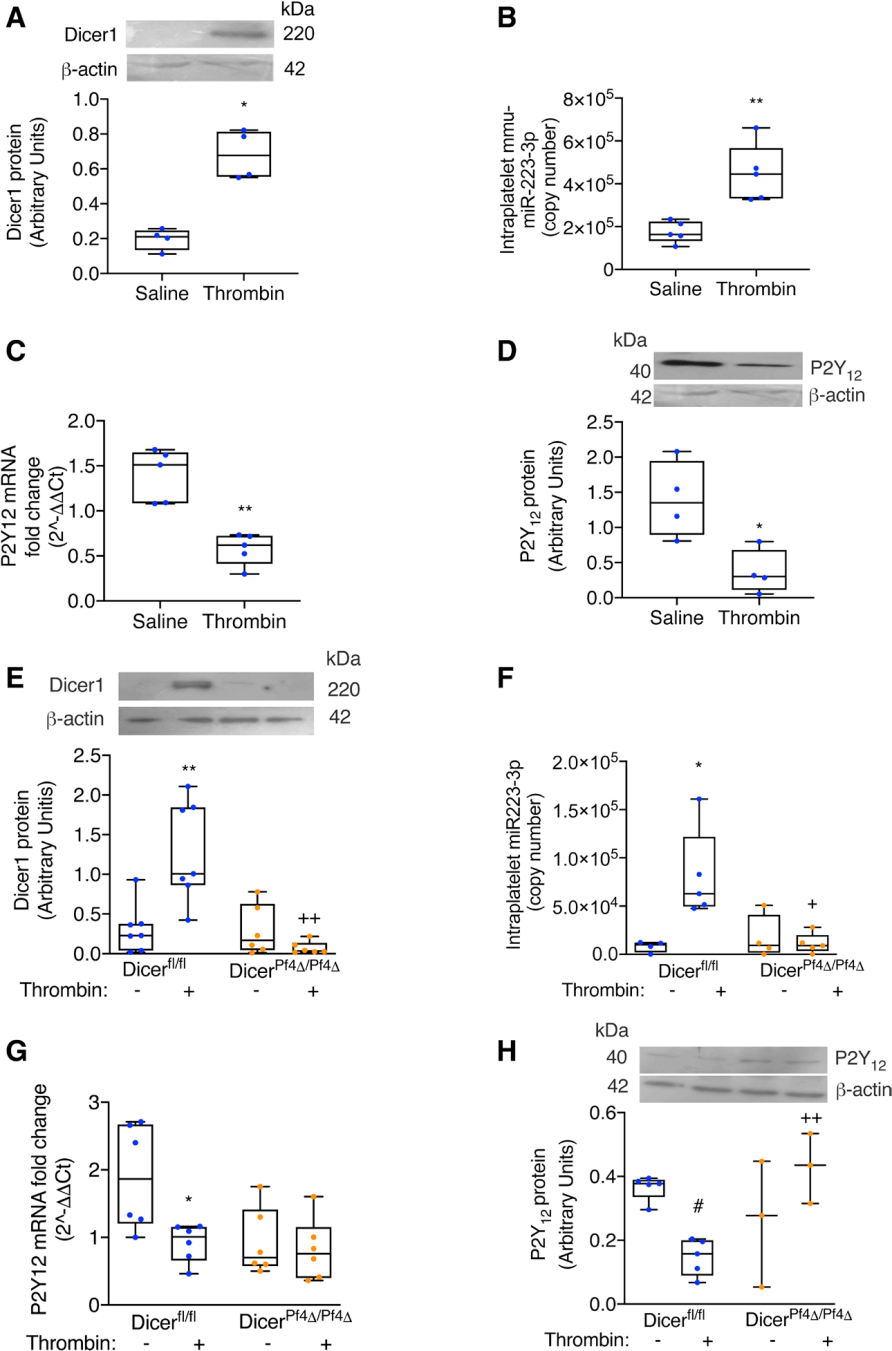
**Thrombin modifies Dicer1 (ribonuclease III enzyme Dicer-1), miR-223-3p (micro RNA 233-3p), P2RY_12_ (purinergic receptor P2Y_12_) mRNA, and P2Y_12_ protein expression in vivo. A**, Western blotting of Dicer1 protein expression in platelets of C57BL/6 mice 60 minutes after injection of thrombin (500 U/kg) or saline. β-actin was used as a loading control. Optical densitometric analysis was performed using the Image J software, and results are expressed in arbitrary units. Results are shown as mean with SD showing individual points; n=4 mice per group. *P=0.0286 Mann-Whitney *U* test. **B**, Absolute quantification of intraplatelet mature mmu-miR-223-3p (murine micro RNA 233-3p) 60 minutes after injection of thrombin or saline in C57BL/6 mice, as measured by real-time polymerase chain reaction (PCR). The expression of mmu-miR-223-3p is reported as a copy number. Results are shown as mean with SD showing individual points; n=5 mice per group. **P=0.0079 Mann-Whitney *U* test. **C**, Expression of P2Y_12_-coding mRNA in platelets of C57BL/6 mice 60 minutes after the injection of thrombin or saline, as measured by real-time PCR. Results are presented as 2^-ΔΔCt^, with B2M (beta-2-microglobulin) and GAPDH serving as housekeeping genes for normalization. Results are shown as mean with SD showing individual points; n=5 mice per group. **P=0.0079 Mann-Whitney *U* test. **D**, Western blotting of P2Y_12_ protein in platelets of C57BL/6 mice 60 minutes after the injection of thrombin or saline. β-actin was used as a loading control. Optical densitometric analysis was performed using Image J software, and results are expressed in arbitrary units. Results are shown as mean with SD showing individual points; n=4 mice per group. *P=0.0286 Mann-Whitney *U* test. **E**, Western blotting of Dicer1 protein expression in platelets of Dicer1^*fl/fl*^ and Dicer1^*Pf4Δ/Pf4Δ*^ mice 60 minutes after the injection of thrombin or saline. β-actin was used as a loading control. Optical densitometric analysis was performed using the Image J software, and results are expressed in arbitrary units. Results are shown as mean with SD showing individual points; n=7 mice per group for Dicer1^*fl/fl*^ and n=6 mice per group for Dicer1^*Pf4Δ/Pf4Δ*^. **P=0.0063 saline vs thrombin for Dicer1^*fl/fl*^ and *P*=0.1375 saline vs thrombin for Dicer1^*Pf4Δ/Pf4Δ*^; the Mann-Whitney *U* test, with Bonferroni correction for multiple comparisons. Comparison between Dicer1^*fl/fl*^ and Dicer1^*Pf4Δ/Pf4Δ*^: ++*P*=0.0013 Dicer1^*fl/fl*^ vs Dicer1^*Pf4Δ/Pf4Δ*^ with thrombin and *P*>0.9999 Dicer1^*fl/fl*^ vs Dicer1^*Pf4Δ/Pf4Δ*^ with saline. The Mann-Whitney *U* test, with Bonferroni correction for multiple comparisons. **F**, Absolute quantification of the intraplatelet mature mmu-miR-223-3p in Dicer1^*fl/fl*^ and Dicer1^*Pf4Δ/Pf4Δ*^ mice 60 minutes after the injection of thrombin or saline, as measured by real-time PCR. The expression of mmu-miR-223-3p is reported as a copy number. Results are shown as mean with SD showing individual points; n=4 mice per group saline and n=5 mice per group thrombin. *P=0.0360 saline vs thrombin for Dicer1^*fl/fl*^ and *P*>0.9999 saline vs thrombin for Dicer1^*Pf4Δ/Pf4Δ*^; the Mann-Whitney *U* test, with Bonferroni correction for multiple comparisons. Comparison between Dicer1^*fl/fl*^ and Dicer1^*Pf4Δ/Pf4Δ*^: +*P*=0.0227 Dicer1^*fl/fl*^ vs Dicer1^*Pf4Δ/Pf4Δ*^ with thrombin and *P*=0.9225 Dicer1^*fl/fl*^ vs Dicer1^*Pf4Δ/Pf4Δ*^ with saline. The Mann-Whitney *U* test, with Bonferroni correction for multiple comparisons. **G**, Expression of P2Y_12_-coding mRNA in platelets of Dicer1^*fl/fl*^ and Dicer1^*Pf4Δ/Pf4Δ*^mice 60 minutes after the injection of thrombin or saline, as measured by real-time PCR. Results are presented as 2^-ΔΔCt^, with B2M and GAPDH serving as housekeeping genes for normalization. Results are shown as mean with SD showing individual points; n=6 mice per group. *P=0.0314 saline vs thrombin for Dicer1^*fl/fl*^ and *P*>0.9999 saline vs thrombin for Dicer1^*Pf4Δ/Pf4Δ*^; the Mann-Whitney *U* test, with Bonferroni correction for multiple comparisons. Comparison between Dicer1^*fl/fl*^ and Dicer1^*Pf4Δ/Pf4Δ*^: *P*>0.9999 Dicer1^*fl/fl*^ vs Dicer1^*Pf4Δ/Pf4Δ*^ with thrombin; *P*=0.0.0543 Dicer1^*fl/fl*^ vs Dicer1^*Pf4Δ/Pf4Δ*^ with saline. The Mann-Whitney *U* test, with Bonferroni correction for multiple comparisons. **H**, Western blotting of P2Y_12_ protein in platelets of Dicer1^*fl/fl*^ and Dicer1^*Pf4Δ/Pf4Δ*^ mice 60 minutes after the injection of thrombin or saline. The β-actin was used as a loading control. Optical densitometric analysis was performed using Image J software, and results are expressed in arbitrary units. Results are shown as mean with SD showing individual points; n=5 mice per group for Dicer1^*fl/fl*^ and n=3 mice per group for Dicer1^*Pf4Δ/Pf4Δ*^. #*P*=2×10^−4^ saline vs thrombin for Dicer1^*fl/fl*^ and *P*=0.5307 saline vs thrombin for Dicer1^*Pf4Δ/Pf4Δ*^; the Mann-Whitney *U* test, with Bonferroni correction for multiple comparisons. Comparison between Dicer1^*fl/fl*^ and Dicer1^*Pf4Δ/Pf4Δ*^: ++*P*=0.0055 Dicer1^*fl/fl*^ vs Dicer1^*Pf4Δ/Pf4Δ*^ with thrombin and *P*=0.5337 Dicer1^*fl/fl*^ vs Dicer1^*Pf4Δ/Pf4Δ*^ with saline. The Mann-Whitney *U* test, with Bonferroni correction for multiple comparisons.

To assess whether thrombin generation in vivo triggers, in platelets, the same chain of events observed in vitro, we injected intravenously a sublethal dose of thrombin in mice and collected blood 60 minutes after injection. This was the time point at which Dicer expression and microRNA-223 expression were increased, and simultaneously, P2Y_12_ mRNA and protein expressions were decreased in the in vitro experiments. We found that intraplatelet Dicer1 (Figure 5A) and mmu-miR-223-3p (murine micro RNA 223-3p) (Figure 5B) increased significantly compared with saline-injected mice, and concomitantly, the expression of P2RY12 mRNA (Figure 5C) and P2Y_12_ decreased (Figure 5D). The regulation of P2Y_12_ expression by microRNA-223 in mice is debated^9,32^; therefore, we assessed whether transfection of murine megakaryocytes with a synthetic mmu-miR-223-3p could decrease P2RY12 mRNA expression. Real-time PCR showed a striking downregulation of murine P2RY12 mRNA upon mmu-miR-223-3p transfection, suggesting that P2RY12 is a target of microRNA-223 also in mice (Figure S18). To test whether the in vivo effect of thrombin on platelet miR-223-3p and P2Y_12_ was dependent on Dicer1 neosynthesis, we performed the same experiments using mice carrying megakaryocyte-specific deletion of Dicer1 (Dicer1^*Pf4Δ/Pf4Δ*^ mice).^33^ As expected, no statistically significant difference was observed regarding platelet Dicer1 increase after thrombin injection in Dicer1^*Pf4Δ/Pf4Δ*^ mice, while it increased in control Dicer1^*fl/fl*^ mice (Figure 5E). Accordingly, no statistically significant difference was observed regarding miR-223-3p increase (Figure 5F), and, consequently, P2RY12 mRNA and P2Y_12_ protein, in Dicer1^*Pf4Δ/Pf4Δ*^mice differently from Dicer1^*fl/fl*^ mice (Figure 5G and 5H).

**Figure 6.**
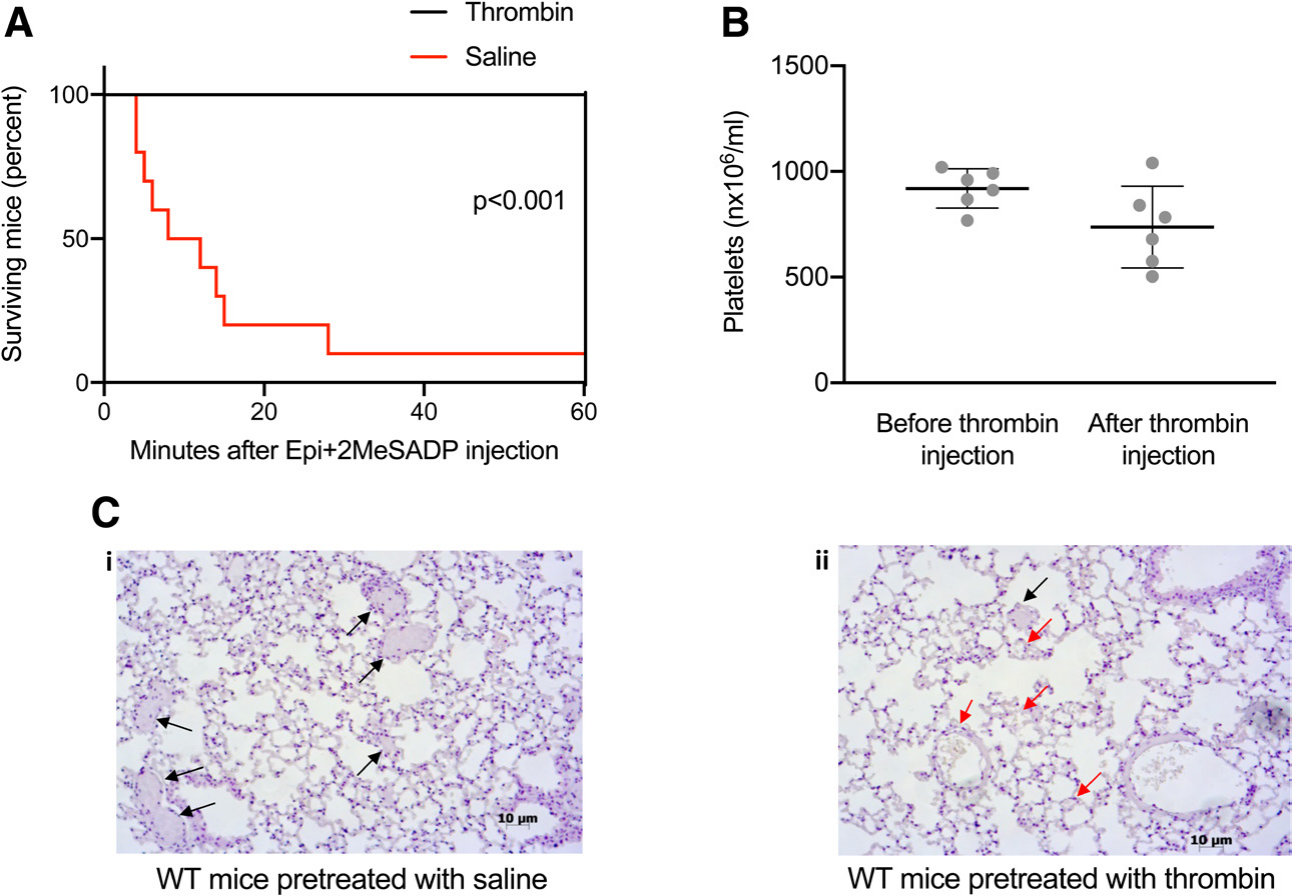
**Injection of a sublethal dose of thrombin prevents platelet pulmonary thromboembolism–induced mortality. A**, Mice were first injected intravenously with a sublethal dose of thrombin (500 U/kg) or saline. After 60 minutes, platelet pulmonary embolism was triggered by intravenous injection of 2-methylthioadenosine 5′-diphosphate (2MeSADP; 20 mg/kg) plus epinephrine (30 mg/kg). N=10 mice per group (*P*<0.001). **B**, Platelet count was assessed before and after thrombin injection in blood anticoagulated with 4% Na citrate. Results are shown as mean with SD showing individual points; n=6 mice per group. *P*=0.1320, the Mann-Whitney *U* test. **C**, Representative images of lung histology stained with hematoxylin and eosin (H&E). Black arrows indicate platelet thrombi on occluded vessels; red arrows indicate open vessels. **i**, Wild-type (WT) mice pretreated with saline or (**ii**) thrombin intravenously and, after 60 minutes, with a mixture of 2MeSADP (20 mg/kg) and epinephrine (30 mg/kg). Scale bar, 10 µm.

To check whether Dicer1-mediated downregulation of P2Y_12_ expression may reduce platelet reactivity and, thus, prevent thrombus formation, we triggered pulmonary embolism using a mix of 2-methylthioadenosine 5′-diphosphate and epinephrine in mice pretreated with a sublethal dose of thrombin, and we, indeed, observed a strikingly reduced mortality compared with mice pretreated with saline (Figure 6A and 6C). Increased survival of the group treated with thrombin was not due to the small, not significant drop of platelet count caused by thrombin injection (about 20%), which was previously shown not to influence thrombus formation in mice^34^ (Figure 6B).

### Dicer1 Neosynthesis and Its Downstream Effects on Gene Expression Are Altered in Platelets From Diabetic Mice

**Figure 7.**
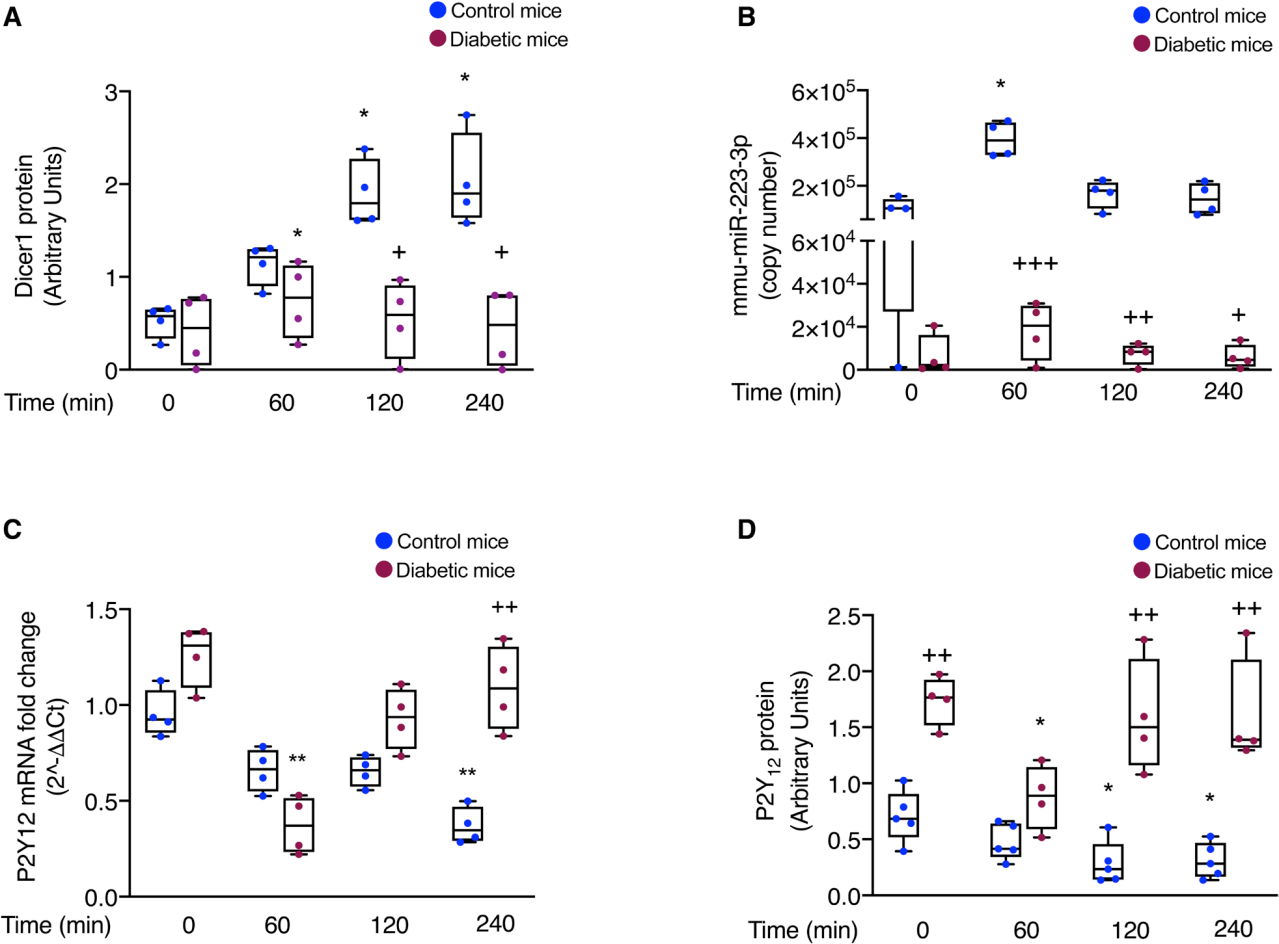
**Thrombin-induced Dicer1 (ribonuclease III enzyme Dicer-1) neosynthesis and downstream events are altered in platelets from diabetic mice. A**, Western blotting of Dicer1 protein in platelets from diabetic and control mice upon stimulation with thrombin (0.1 U/mL). β-actin was used as a loading control. Optical densitometric analysis was performed using the Image J software, and results are expressed in arbitrary units. Results are shown as mean with SD showing individual points; n=4 mice per group. For control mice: *P=0.0185 time 120 vs time 0, *P=0.0185 time 240 vs time 0, and *P*=0.8199 time 60 vs time 0. For diabetic mice: *P=0.0411 time 60 vs time 0 and *P*>0.9999 time 120 and time 240 vs time 0. The Friedman test was followed by the Dunn multiple comparisons test. Comparison between control mice and diabetic mice: +*P*=0.0105 controls vs type 2 diabetes (T2DM) at time 120, +*P*=0.0115 controls vs T2DM at time 240, *P*>0.9999 controls vs T2DM at time 0, and *P*=0.5804 controls vs T2DM at time 60. The Mann-Whitney *U* test, with Bonferroni correction for multiple comparisons. **B**, Absolute quantification of the intraplatelet mature mmu-miR-223-3p (murine micro RNA 233-3p) over time upon stimulation with thrombin (0.1 U/mL), as measured by real-time polymerase chain reaction (PCR) in platelets from diabetic mice compared with control mice. The expression of mmu-miR-223-3p is reported as a copy number. Results are shown as mean with SD showing individual points; n=4 mice per group. For control mice: *P=0.0185 time 60 vs time 0, *P*=0.8199 time 120 vs time 0, and *P*>0.9999 time 240 vs time 0. For diabetic mice: *P*=0.8199 time 60 vs time 0 and *P*>0.9999 time 120 and 240. The Friedman test was followed by the Dunn multiple comparisons test. Comparison between control mice and diabetic mice: *P*=0.1622 controls vs T2DM at time 0, +++*P*=2.3×10^−4^ controls vs T2DM at time 60, ++*P*=0.0074 controls vs T2DM at time 120, and +*P*=0.0220 controls vs T2DM at time 240. The Mann-Whitney *U* test, with Bonferroni correction for multiple comparisons. **C**, Expression of P2Y_12_ (purinergic receptor P2Y_12_)-coding mRNA over time upon platelet stimulation with thrombin (0.1 U/mL), as measured by real-time PCR in platelets from diabetic mice compared with control mice. Results are presented as 2^-ΔΔCt^, with B2M (beta-2-microglobulin) and GAPDH serving as housekeeping genes for normalization. Results are shown as mean with SD showing individual points; n=4 mice per group. For control mice: *P*=0.3010 time 60 vs time 0, *P*=0.3010 time 120 vs time 0, and **P=0.0030 time 240 vs time 0. For diabetic mice: **P=0.0077 time 60 vs time 0, *P*=0.3960 time 120 vs time 0, and *P*>0.9999 time 240 vs time 0. The Friedman test was followed by the Dunn multiple comparisons test. Comparison between control mice and diabetic mice: *P*=0.0635 controls vs T2DM at time 0, *P*=0.0635 controls vs T2DM at time 60, *P*=0.0635 controls vs T2DM at time 120, and ++*P*=0.0039 controls vs T2DM at time 240. The Mann-Whitney *U* test, with Bonferroni correction for multiple comparisons. **D**, Western blotting of P2Y_12_ protein over time upon platelet stimulation with thrombin (0.1 U/mL) in platelets from diabetic mice compared with control mice. β-actin was used as a loading control. Optical densitometric analysis was performed using Image J software, and results are expressed in arbitrary units. Results are shown as mean with SD showing individual points; n=4 mice per group. For control mice: *P=0.0219 time 120 vs time 0, *P=0.0261 time 240 vs time 0, and *P*=0.2319 time 60 vs time 0. For diabetic mice: *P=0.0368 time 60 vs time 0, *P*=0.9957 time 120 vs time 0, and *P*=0.9895 time 240 vs time 0. The Friedman test was followed by the Dunn multiple comparisons test. Comparison between control mice and diabetic mice: ++*P*=0.0010 controls vs T2DM at time 0, *P*=0.1322 controls vs T2DM at time 60, ++*P*=0.0042 controls vs T2DM at time 120, and ++*P*=0.0033 controls vs T2DM at time 240. The Mann-Whitney *U* test, with Bonferroni correction for multiple comparisons.

Upon intravenous thrombin injection, platelet Dicer1 and miR-223-3p increased in diabetic mouse platelets, but then they more rapidly decreased, differently from control mice (Figure 7A and 7B). In diabetic mice, thrombin injection induced an initial downregulation of platelet P2RY12 mRNA and P2Y_12_ protein, but then their expression increased again going back to basal levels after 120 minutes, differently from control mice (Figure 7C and 7D).

## Discussion

Our results unravel a self-regulatory mechanism of platelet reactivity to ADP initiated by the exposure to thrombin and due to the de novo synthesis of Dicer1, which, in turn, induces modifications of platelet microRNA, mRNA, and protein expression, a process impaired in T2DM. In particular, we showed that thrombin stimulation leads to the maturation of pre-miR-223 into miR-223-3p, to the downregulation of P2RY12 target mRNA, and to reduced expression and function of P2Y_12_, the purinergic receptor mediating ADP-induced aggregation.

We found that a derangement of this self-regulatory mechanism occurs in diabetes, probably contributing to the platelet hyperreactivity and in vivo activation typical of these patients.^35,36^ In fact, although thrombin triggered microRNA-223 maturation also in T2DM platelets, this phenomenon was short-lasting, and P2RY12 mRNA rapidly returned to normal compared with healthy control platelets, leading to a premature restoration of receptor levels and function, possibly facilitating platelet hyperresponsiveness to ADP.

Thus, Dicer1 plays a crucial role in the modulation of the platelet function phenotype by triggering the maturation of specific microRNAs suppressing mRNA transcription. This self-regulatory mechanism may serve to reduce the reactivity of platelets, which have already been exposed to low concentrations of thrombin, to further stimuli, to prevent unwanted thrombus formation. Indeed, we showed that these phenomena occur in vivo in platelets of wild-type mice, but not those from Dicer^-/-^ mice, after the intravenous injection of sublethal doses of thrombin. A derangement of this self-regulatory mechanism in patients with T2DM may partly account for the hyperreactivity of their platelets to ADP.

The presence of Dicer1 in human platelets was previously reported; however, its scarcity seemed inconsistent with the abundance of microRNAs in platelets.^28^ We confirm that, indeed, Dicer1 is present in very small amounts in the cytoplasm of resting human platelets, but we show that upon thrombin stimulation, new protein is synthesized, which binds to the cytoskeleton. The latter finding is in line with previous observations showing that other platelet cytoplasmic proteins regulating gene expression, like the translation initiation factor eIF4E (eukaryotic translation initiation factor 4E), redistribute to the cytoskeleton upon activation together with mRNAs,^37^ a process required for signal-dependent protein synthesis.^38^

Dicer1 neosynthesis in platelets is rapid, with a strong increase observed already after 3 minutes. A rapid neosynthesis of Dicer1 is consistent with the translation of a constitutively expressed mRNA with no requirement for premRNA maturation and with the biology of platelets as rapidly responding cells. Indeed, platelet stimulation with thrombin phosphorylates eIF4E, an initiator of protein synthesis, within 1 minute.^37^

Interestingly, Dicer1 synthesis in platelets was triggered by thrombin and ADP, but not by collagen, suggesting a selectivity of different signal transduction pathways in regulating mRNA translation. This is not surprising because the receptors of both thrombin and ADP belong to the family of GPCRs (G-protein–coupled receptors), and G-proteins are known to be involved in protein synthesis, while ITAM (immunoreceptor tyrosine-based activation motif)-coupled receptors, such as the collagen receptor GPVI, have not been directly linked to mRNA translation.^39^

Neosynthesis of Dicer1 was platelet-specific because, in our experiments, leukocyte contamination was negligible; it was confirmed by the incorporation of ^35^S-radiolabeled methionine in the newly synthesized protein; and it was blocked by protein synthesis inhibitors. Dicer1, thus, adds to the growing list of proteins synthesized by platelets with an mRNA relying on signal-dependent events for translation.^40^ Its synthesis is controlled by the mTOR and Mnk1 pathway; accordingly, Dicer1 mRNA 5’-UTR has a secondary structure with a free energy of 101.5 kcal/mol, typical of transcripts that depend on mTOR for translation.^41^ We observed a decrease in Dicer1 mRNA 4 hours after thrombin stimulation, consistent with the progressive decay of the platelet transcriptome in the absence of nuclear transcription.^42,43^

A role for Dicer1 in microRNA processing has previously been shown in murine platelets, using megakaryocyte-selective knockdown mice for Dicer1,^33^ and also in human platelets, but only using exogenous pre-microRNAs and not endogenous precursors,^1^ while no studies have assessed the role of Dicer1 in the maturation of platelet microRNA upon activation.

Here, we show for the first time that Dicer1 neosynthesis associates with an increase in miR-223-3p in platelets, already evident 15 minutes after thrombin stimulation and persisting for up to 240 minutes, while pre-miR-223 shows an opposite trend. Thrombin-triggered microRNA changes were blocked by the inhibition of Dicer1 neosynthesis and by the removal of Dicer1 by immunoprecipitation. Taken together, our results show that, upon activation, platelets convert a reservoir of pre-microRNAs received from megakaryocytes into mature microRNAs by neosynthesizing Dicer1.

It was previously reported that P2Y_12_ expression may be subjected to microRNA-223 control in human platelets.^1^ We show here that upon thrombin stimulation, P2RY12 mRNA decreases in platelets, a reduction inversely related to Dicer1 neosynthesis, and this phenomenon associates with a diminished expression of P2Y_12_. These data are in agreement with recent observations showing a significant reorganization of the platelet miRNome and proteome during activation.^13^ The fast decrease implies a rapid turnover of P2Y_12_, and it is known that, upon activation, platelet receptors, including P2Y_12_, are internalized and shuttled from the plasma membrane to endosomes where they may undergo lysosomal degradation.^44^

The decrease in P2Y_12_ was associated with a reduction of its function, with impaired adenylylcyclase-inhibitory activity, which was restored by the inhibition of Dicer neosynthesis.

The decrease in platelet P2Y_12_ expression after thrombin activation may represent a self-regulatory mechanism to downregulate subsequent activation. Indeed, platelets not terminally activated continue to circulate and function in vivo.^45^ Therefore, while a burst of thrombin generation at a site of vessel wall damage irreversibly activates platelets in synergy with ADP,^46,47^ chronic low-level thrombin generation^48–50^ may downregulate P2Y_12_ expression, thus reducing platelet reactivity to prevent unwanted thrombus formation. Therefore, we simulated the endogenous generation of low levels of thrombin by injecting mice intravenously with a sublethal dose of thrombin. We indeed observed Dicer1 neosynthesis, an increase in microRNA-223, and downregulation of P2RY12 mRNA and P2Y_12_ in platelets, phenomena abrogated in Dicer1-deficient mice. Our results also show that a derangement of this self-regulatory mechanism occurs in diabetes, in which thrombin-triggered microRNA-223 maturation was transient, and P2RY12 mRNA rapidly returned to normal.

How mature P2RY12 mRNA may increase in the absence of a nucleus is still unclear. This phenomenon was independent from transcription because the increase in P2RY12 mRNA was still detected after incubation with actinomycin D excluding the possibility of new transcription from contaminating genomic DNA in our preparation. We used exon-spanning primers to measure P2RY12 mRNA expression; therefore, it can not be excluded that immature premRNA is matured by splicing.^51^ However, we could not detect any P2RY12 premRNA in platelets (data not shown). Insight into the mechanisms regulating gene expression and protein synthesis in platelets is rapidly evolving,^52^ and other still unknown mechanisms may be discovered, which may explain our findings. The calpain inhibitor calpeptin^7,9^ re-established Dicer1 neosynthesis and microRNA-223 maturation, demonstrating that these phenomena in T2DM platelets were dependent on the degradation of Dicer1 by calpain. This was further demonstrated in vivo; indeed, thrombin injection in mice stimulated Dicer1 neosynthesis with consequent downregulation of P2Y_12_ expression, but this phenomenon was abrogated in Dicer1^*Pf4Δ/Pf4Δ*^mice and defective in diabetic mice. Moreover, we proved that mice injected with a sublethal dose of thrombin survive to pulmonary thromboembolism induced by MeSADP (2-methylthio-ADP) and epinephrine. Of course, this does not exclude that additional mechanisms, besides Dicer increase and microRNA-223 upregulation, modulate P2Y_12_ expression in T2DM.

Another potential function of platelet microRNAs matured through neosynthesized Dicer1 is the transcellular regulation of gene expression. In fact, it is known that platelets transfer RNAs and microRNAs to other cells, in this way modulating the phenotype of the latter,^11,31,53,54^ and we here show that activated platelets transfer their miR-223-3p to PMPs. However, differently from Ago2, which is found in PMPs bound to microRNAs,^31^ we failed to detect Dicer1. This is compatible with proteomic studies of PMPs, showing that only a portion of the platelet proteins, including cytoplasmic proteins, are sorted to PMPs,^55,56^ and with the finding that neosynthesized Dicer1 binds to the platelet cytoskeleton. Therefore, it can be speculated that Dicer1 is not sorted to PMPs because it is functional to the maturation of microRNAs inside platelets.

In summary, our study shows, for the first time, that thrombin-stimulated platelets synthesize Dicer1 upon activation in vitro and in vivo, and this event regulates the expression of microRNAs and their target mRNAs. Moreover, we show that a derangement of these mechanisms in T2DM leads to platelet hyperreactivity. In conclusion, platelets regulate their own gene expression through the neosynthesis of Dicer1, adding a further level of complexity to the posttranscriptional regulation of protein synthesis in these anucleated cells.

## Article Information

### Acknowledgments

The authors thank Dr Gabriele Perriello (Diabetology Clinic, Internal Medicine, Endocrine and Metabolic Sciences, Perugia Hospital) for help with enrollment of patients with type 2 diabetes (T2DM); Prof Ferdinando Mannello and Dr Caterina Ciacci, University of Urbino, for help with confocal microscopy; Prof J.E. Jr Italiano, Harvard Medical School, Cambridge, MA, for the rabbit anti-human beta1-tubulin antibody; Prof C. Van Geet and Prof K. Freson, University of Leuven, Leuven, Belgium, for providing the MEG-01 cell line; Dr Pierluigi Orvietani, University of Perugia, for help with ultracentrifugation of samples; and Prof Matthew Rondina, University of Utah School of Medicine, Salt Lake City, UT, for constructive discussion of the results.

### Author Contributions

P. Gresele designed the study. L. Bury, E. Piselli, G. Manni, S. Momi, G. Ciarrocca Taranta, and J.W. Rowley performed the experiments. C. Fanelli and M. De Fano provided patients with T2DM to the study. L. Bury, E. Piselli, G. Manni, G. Guglielmini, and J.W. Rowley analyzed data. L. Bury, E. Piselli, G. Manni, and P. Gresele wrote the manuscript. J.W. Rowley, A.S. Weyrich, and P. Gresele critically reviewed the manuscript. All authors read and approved the manuscript.

### Sources of Funding

This work was supported in part by grants to P. Gresele from MIUR-FIRB (protocol RBFR12W5V5_004), from H2020-FETOPEN-2014-CSA (protocol 665564), and from Fondazione CARIPLO (grant 2018) and by a fellowship to L. Bury by the Fondazione Umberto Veronesi.

### Disclosures

None.
